# Frequency-Dependent Surface-Discharge Lifetime and PRPD Failure Signatures of Polyimide Insulation Under High-Frequency Sinusoidal Voltage

**DOI:** 10.3390/polym18161987

**Published:** 2026-08-15

**Authors:** Bei Li, Tianrun Qi, Qingmin Li

**Affiliations:** 1 School of Engineering, The University of Edinburgh, Edinburgh EH9 3FB, UK; 2 State Key Laboratory of Alternate Electrical Power System with Renewable Energy Sources, North China Electric Power University, Beijing 102206, China

**Keywords:** polyimide, surface discharge, high-frequency transformer, solid-state transformer, partial discharge, PRPD pattern, insulation lifetime, failure precursor

## Abstract

Polyimide (PI) film is a widely used insulation material in high-frequency power equipment, yet its frequency–lifetime relation and terminal discharge features have rarely been resolved together over a complete ageing trajectory. This paper reports three contributions. First, a condition-specific frequency–lifetime relation for PI surface insulation is presented, in which the discharge inception voltage is frequency-invariant while the flashover voltage and the surface lifetime both fall with frequency. Second, a stage-resolved discharge morphology derived from phase-resolved patterns is proposed, in which a late-stage finger-like cluster serves as a candidate flashover precursor, complemented by a count-aware, time-normalised discharge activity proxy. Third, a space-charge interpretation linking the per-cycle charge injection budget growth at high frequency to the suppressed inter-cycle dissipation, jointly accounting for the lifetime relation and the non-dendritic damage morphology, is presented. The fitted lifetime relation provides a quantitative planning basis over the tested 10–40 kHz grid for insulation design of high-frequency power equipment, and the finger signature provides a candidate device-level morphological criterion for online monitoring.

## 1. Introduction

High-frequency power transformers (HFPTs), the magnetic heart of solid-state transformers (SSTs), compress weight and volume by raising the operating frequency into the 10–50 kHz band, and pay for it with electrical stress of a kind conventional insulation systems were never qualified for, characterised by high repetition rate, fast polarity reversal and elevated thermal stress [1,2,3]. Beyond voltage amplitude, repetitive-pulse lifetime experiments identify voltage rise rate as a distinct ageing acceleration factor [4], while polymer-insulation lifetime has also been modelled under combined electrical and thermal factors [5]. Their compact gas–solid insulation systems rely heavily on polymer films, above all polyimide (PI), prized for thermal endurance, mechanical robustness and dielectric strength [6]. Pulsed-electroacoustic measurements on PI under sinusoidal excitation up to 500 Hz further show that waveform and repetition frequency reorganise bulk-charge accumulation and the associated internal-field distortion [7]. Along the gas–solid interfaces of such systems, surface discharge is a leading failure path: it progressively erodes the polymer surface and can terminate in flashover [8,9].

The behaviour of PI surface discharge under genuinely high-frequency sinusoidal stress has attracted a sequence of recent studies. Its frequency, temperature and pressure dependence has been mapped through combined experiments and field simulation [10], and stage-wise transformation has been characterised at fixed drive frequency [11]. Parallel work addresses material modification and multiscale degradation [12,13,14]. What remains unresolved is a condition-specific frequency–lifetime relation combined with quantitatively defined terminal-discharge features over the complete ageing trajectory. Addressing this requires systematic constant-stress ageing experiments across a frequency series, with the discharge statistics resolved over the whole trajectory rather than at snapshots.

This paper reports such an experiment. PI film specimens were stressed under 10, 20, 30 and 40 kHz sinusoidal voltage in a needle–rod system with wide-band pulse-current detection. The complete trajectories that reached flashover were reduced to phase-resolved partial-discharge (PRPD) statistics divided into early, middle and late stages. The contributions are threefold. First, a quantitative frequency–lifetime relation for PI surface insulation reports the inception voltage as frequency-invariant while both the flashover voltage and the surface lifetime are not (Section 3). Second, a stage-resolved PRPD morphology study identifies and quantifies a finger-like cluster signature that precedes flashover, which is sharpest at 20 kHz, and complements the geometric signature with a count-aware cumulative discharge activity proxy per unit time that is derived from the stage statistics and ranked opposite to lifetime (Section 4). Third, a space-charge interpretation links the per-cycle charge injection budget growth at high frequency to the suppressed inter-cycle dissipation, accounting jointly for the lifetime relation and the non-dendritic damage morphology (Section 5).

## 2. Materials and Methods

### 2.1. Samples and Electrode System

Each specimen was a 175 μm polyimide film mounted flat on a 5 cm-thick epoxy substrate; the substrate provides the vertical withstand required by the arrangement and keeps stray capacitance to ground low. All specimens came from the same film batch and retained the same 175 μm thickness so that drive frequency remained the isolated comparison variable. Prior to installation, films underwent alcohol cleaning followed by 24 h of oven drying at 60 °C. The stressing head modelled the sharp-point defect of a gas–solid insulation system through a needle–rod arrangement: the needle electrode, inclined at 15° to the film surface, rested on the specimen, with the rod ground electrode placed 2 cm across the surface. The needle–rod geometry was selected to provide a dominant tangential field along the film while limiting the strong normal-field component that a needle–plane arrangement would impose. The 15° angle was held fixed throughout the frequency comparison. The reported surface-discharge and flashover endpoints refer to the visible needle-to-rod path on the exposed PI surface; the epoxy remained underneath the film as its unexposed supporting substrate. The substrate permittivity contributes to the field distribution of this composite arrangement. Its material, thickness and position were held fixed at every frequency, providing a controlled basis for comparison along the frequency axis.

### 2.2. High-Frequency Stress and Discharge Detection

**Figure 1:**
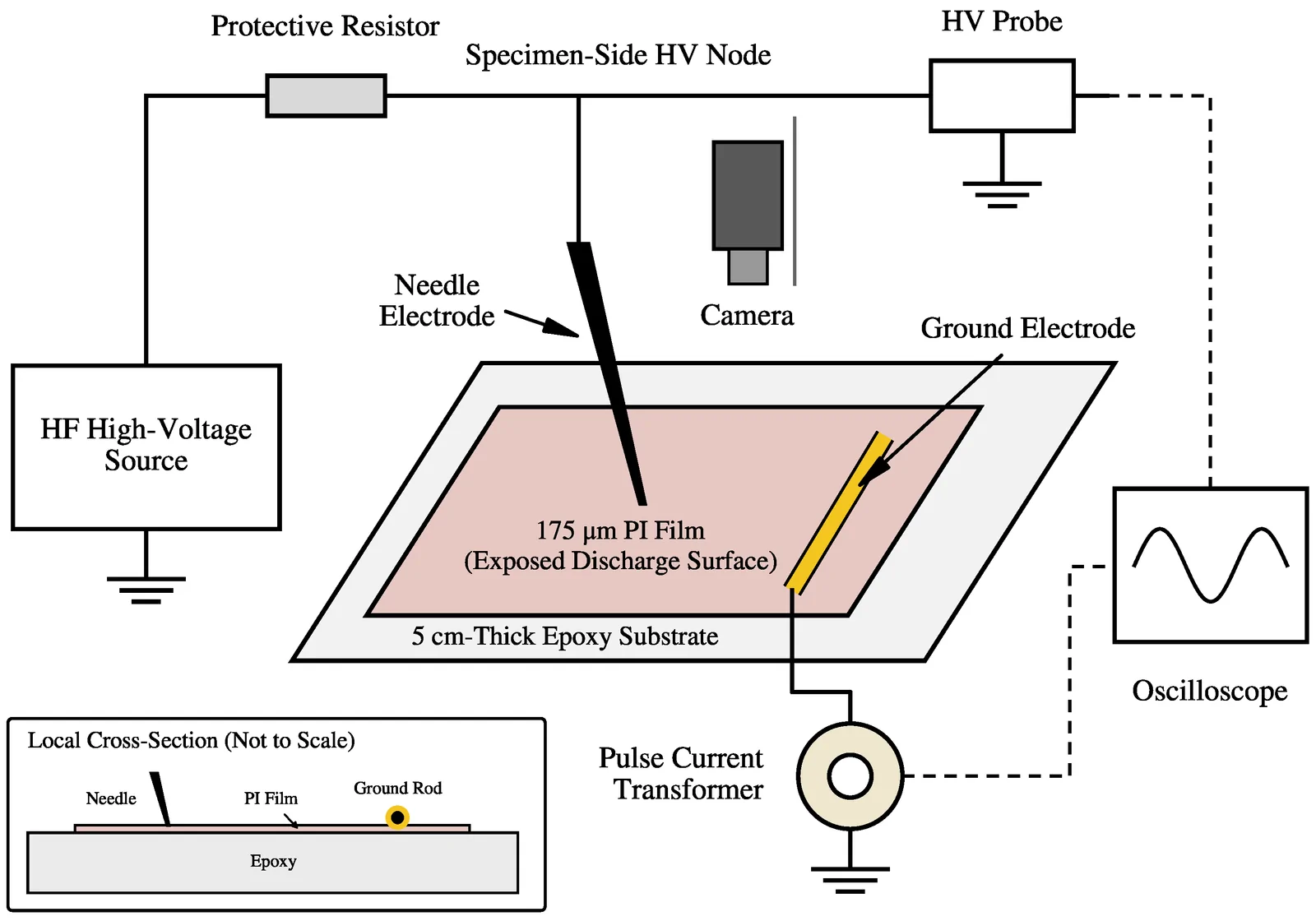
Schematic of the surface discharge measurement arrangement: high-frequency high-voltage source, protective resistor, needle electrode resting on the exposed polyimide (PI) film surface, rod ground electrode, epoxy substrate, wide-band pulse current transformer in the ground return, high-voltage probe at the specimen-side node, oscilloscope and optical camera. The cross-section inset distinguishes the PI film from its underlying epoxy support.

Each ageing run recorded two co-digitised electrical channels through a LabVIEW acquisition chain: discharge current and applied voltage. A camera above the needle recorded the optical development of the discharge in parallel. Discharge pulses returned via a wide-band current transformer (ETS-93686; 300 kHz to 100 MHz bandwidth) on the ground path. Applied voltage was measured at the specimen-side node downstream of the protective resistor using a Tektronix P6015A high-voltage probe (Tektronix, Beaverton, OR, USA; 1000:1) and co-digitised with the discharge channel on a four-channel YOKOGAWA DL6154 oscilloscope (Yokogawa Electric, Tokyo, Japan). The acquisition rate was 625 MS/s at 10 and 20 kHz and 1.25 GS/s at 30 and 40 kHz; the oscilloscope has a 10 GS/s maximum sampling rate and a 1.5 GHz analogue bandwidth. Excitation came from a high-frequency high-voltage source with continuously adjustable amplitude across 0 to 30 kV peak to peak and frequency across 10 to 50 kHz. Figure 1 sketches the arrangement.

### 2.3. Test Protocol

Each of the four frequencies (10, 20, 30 and 40 kHz) received two independent protocols. Inception and flashover voltages were obtained from a continuous ramp protocol at 200 V/s, with inception recorded when discharge first appeared and flashover recorded at surface bridging, applied over five runs per frequency; the values reported below are the corresponding five-run means. Surface lifetime was obtained from a constant-stress protocol at 12 kV. This level was above the approximately 3.8 kV inception voltage but below the 16.1–18.4 kV flashover-voltage range measured over the four frequencies, so that surface discharge could develop without immediate flashover. At each frequency, a fresh specimen was subjected to the constant-stress protocol with the full discharge record acquired; elapsed time to surface flashover defined the surface lifetime, with the 10 kHz observation right-censored at the predefined 10 h endpoint.

### 2.4. Signal Conditioning and PRPD Reduction

Pulse trains were processed offline in three stages. Denoising used an eight-level sym8 wavelet decomposition with per-band thresholding; peak extraction over successive windows retained one discharge event per window; a bipolar-discharge test then suppressed unipolar artefacts on the premise that genuine discharges appear as bipolar events while narrowband interference does not. Denoising preserved 93.56 % of the raw signal energy, which confirmed effective interference rejection at minimal signal loss. The event stream was reduced to PRPD patterns thereafter. Stage-resolved statistics partitioned each ageing trajectory into early, middle and late stages by the observed discharge-development progress. For each uncensored trajectory, normalised progress was defined as p=100t/L, where *t* is elapsed time from surface-discharge inception and *L* is that trajectory’s measured time to flashover; each stage was represented by 400 groups of typical waveforms comprising three to five thousand discharge pulses. The late-stage window quantification (Section 4) scores the 320–360° window of the nine recorded pattern panels by its share of the pattern’s occupied area and by the 99th-percentile amplitude of that area. For each panel, the occupied-area share was calculated relative to the total occupied pattern area in that panel, while the amplitude reach was defined by the 99th percentile within the fixed phase window. The same window and definitions were applied to all nine panels without frequency- or stage-specific adjustment.

## 3. Frequency–Lifetime Results

**Figure 2:**
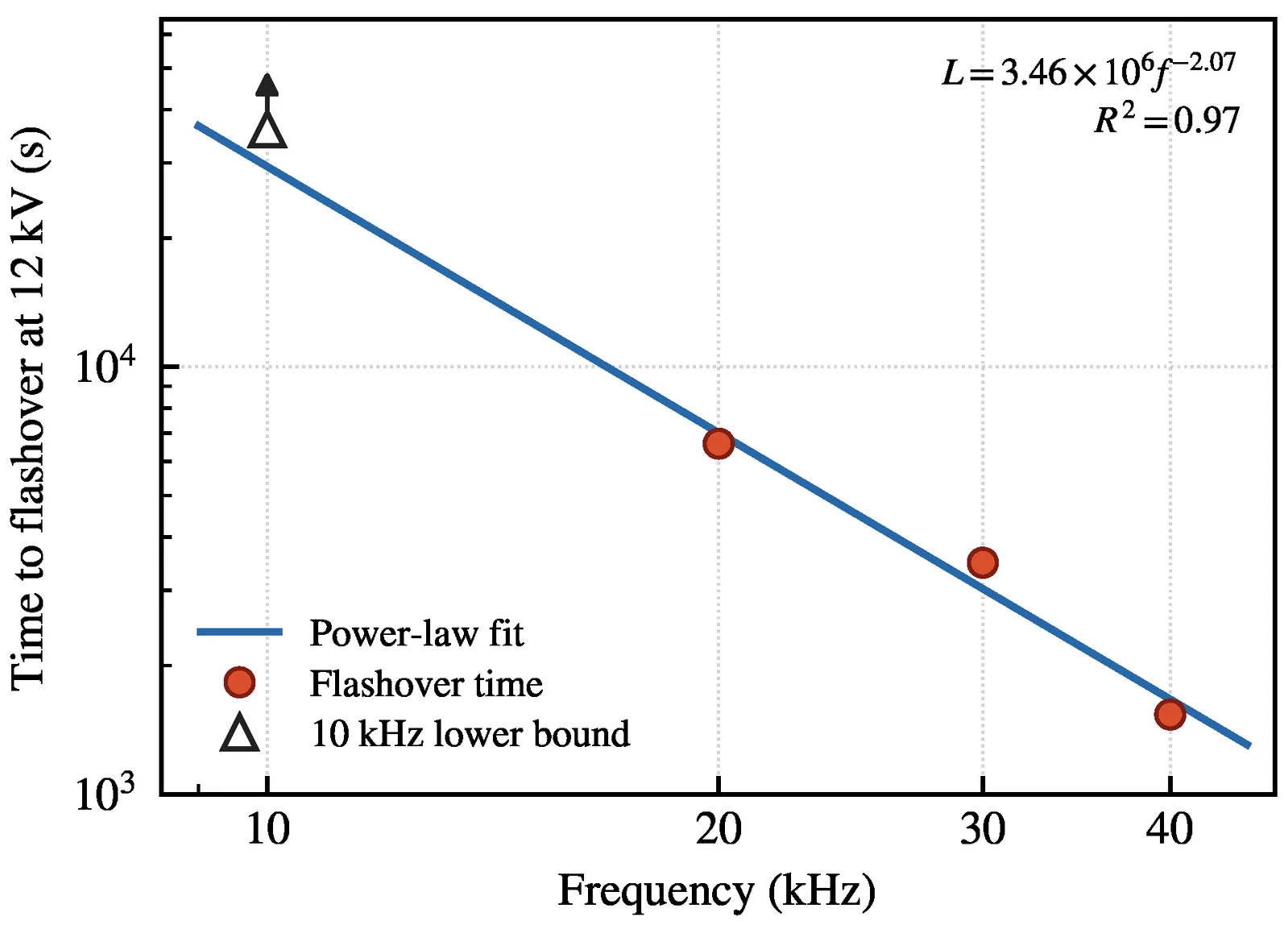
Time to flashover at 12 kV constant stress against frequency (log–log). The line is the least-squares power law over the three flashover runs; the 10 kHz run is censored (no flashover within 10 h; shown as the open triangle, with the upward arrow marking a lower bound) and lies above the extrapolation.

Table 1 carries three organising facts about the frequency dependence. First, the discharge inception voltage remains at about 3.8 kV at every frequency: inception is set by the local field at the needle tip and by the availability of seed electrons, and the observed threshold remains stable under the fixed geometry and voltage protocol. Second, the flashover voltage moves downward, from 18.4 kV at 10 kHz to 16.1 kV at 40 kHz: surface flashover is ageing-mediated, and higher frequency feeds the ageing faster. Third, and of most weight for design practice, the constant-stress lifetime declines as an inverse power of frequency. A log–log least-squares fit to the three measured lifetimes returns $L=3.46\times 10^{6}\;f^{-2.07}$ (with *f* in kHz and *L* in s) at $R^{2}=0.97$ (Figure 2); the pairwise exponents span 1.6 to 2.8, bracketing two, and a strict inverse law is inconsistent with the observed trend because the product Lf decreases monotonically from $1.32\times 10^{5}$ through the three points down to $0.61\times 10^{5}$. At 10 kHz, no surface flashover occurred within the predefined ten-hour test window. This censored run lies above the extrapolated fit (8.2 h), so the decay between 10 and 20 kHz is at least as steep (pairwise exponent ≥2.4), consistent with an inter-cycle interval at 10 kHz long enough for the injected surface charge to dissipate before the next half-cycle reinforces it (Section 5). For an SST designer, the message is blunt: with an exponent of about two, doubling the operating frequency cuts the surface-insulation life at equal stress by a factor of about four (6608 s at 20 kHz against 1536 s at 40 kHz), so frequency increases must be accompanied by either derated field strength or reinforced creepage design.

## 4. Stage-Resolved PRPD Morphology

### 4.1. Magnitude and Repetition Statistics

**Figure 3:**
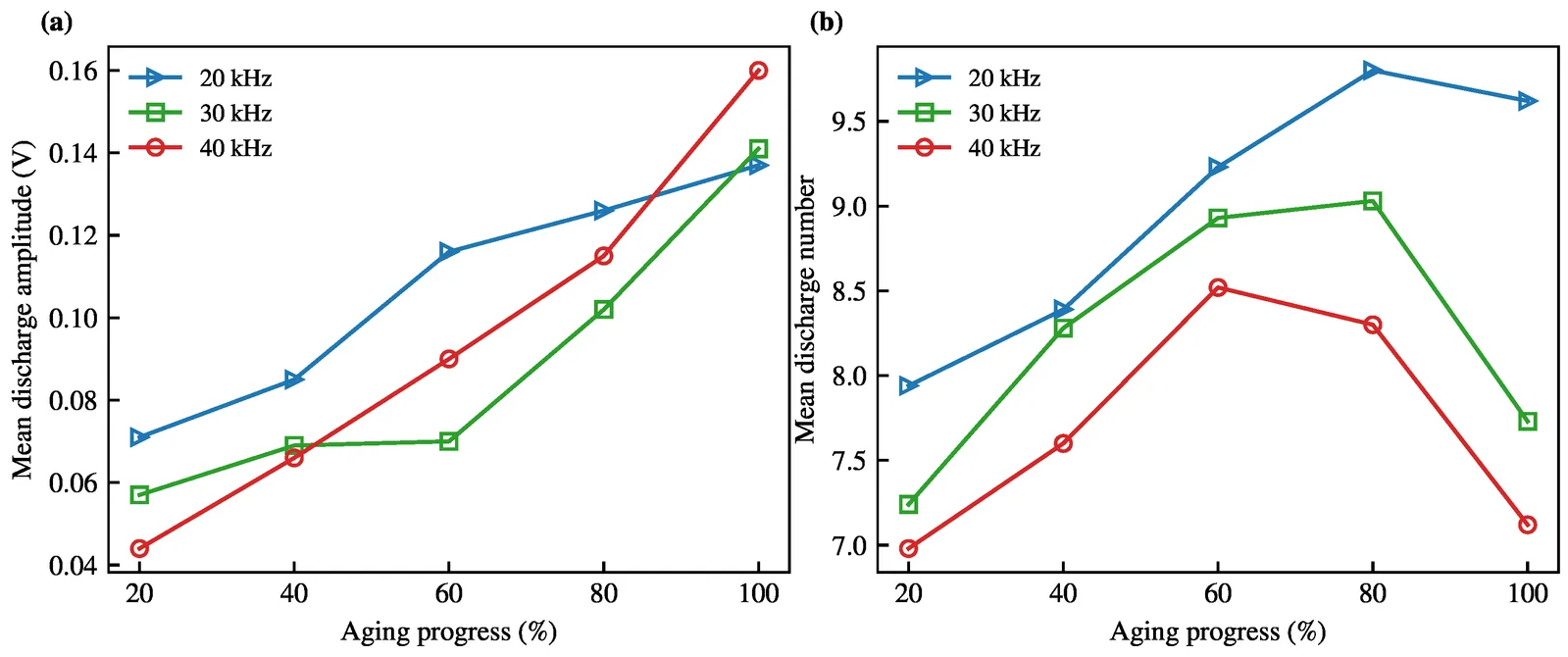
Stage statistics of the discharge record at 20, 30 and 40 kHz against ageing progress: (**a**) mean discharge amplitude; (**b**) mean repetition number per observation window. Amplitude values refer to the high-frequency current-transformer (HFCT) detector-channel output voltage, not the applied high voltage. The 10 kHz run, which did not reach flashover, provides no complete ageing trajectory.

Across the ageing trajectory at each of the three frequencies that reached flashover, the mean discharge magnitude rises monotonically, spanning 0.04 to 0.16 V (detector output) from the 20% point to the 100% point of the lifetime, while the mean discharge repetition number per observation window first rises and then falls, peaking at the 60–80% point (ranges 6.9–9.8 across frequencies). The crossover of these two statistics, with fewer but stronger discharges, marks the transition from distributed surface activity to a concentrating pre-flashover channel (Figure 3).

### 4.2. Stage-Dependent Finger Signature

**Figure 4:**
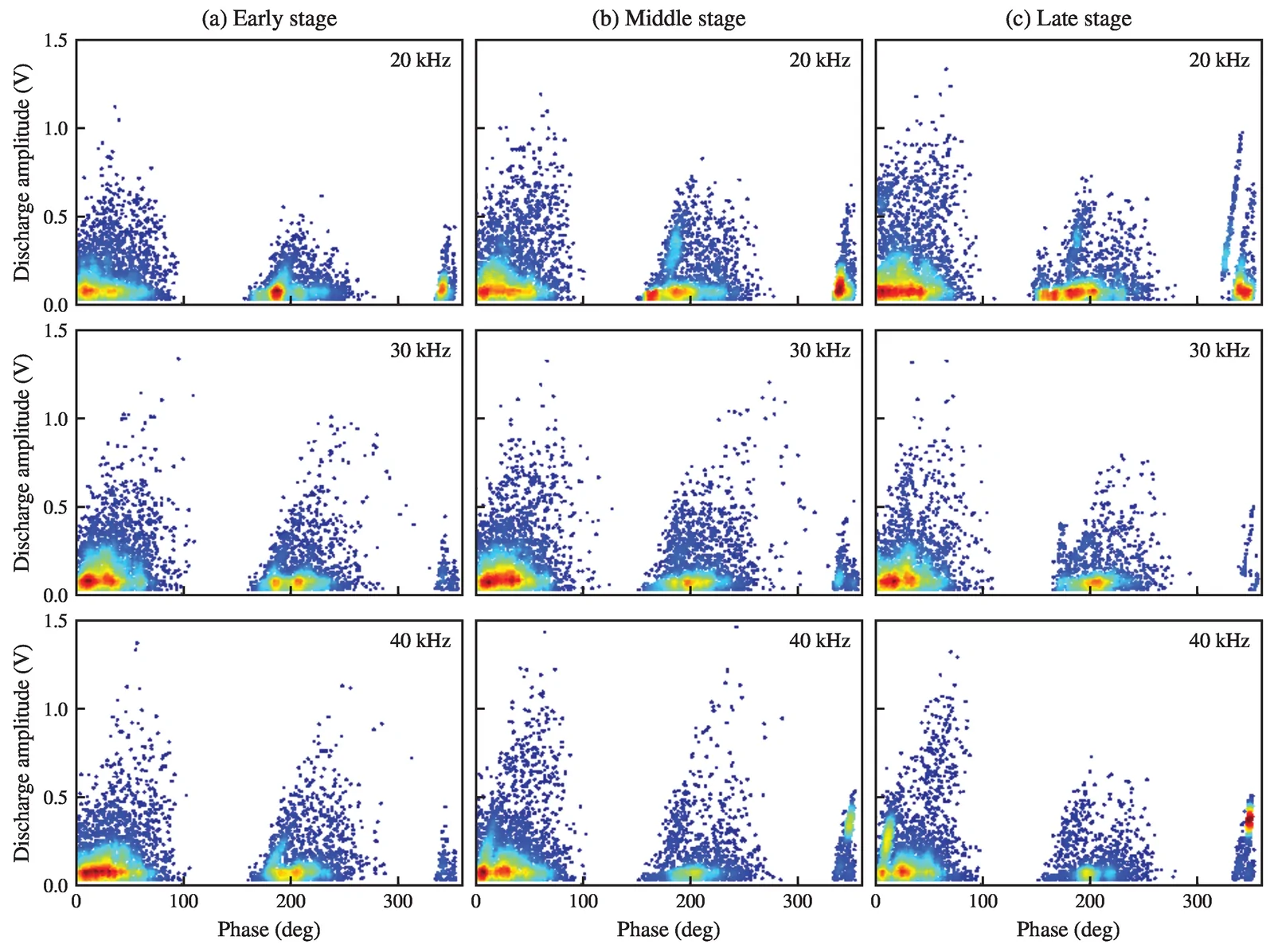
Phase-resolved discharge patterns over the three ageing stages (columns) at 20, 30 and 40 kHz (rows). Finger-like clusters in the 320–360 degree window are most articulated in the late-stage column at 20 kHz; at 40 kHz an elongated form is already visible at mid stage, and at 30 kHz the late-stage form stays low in amplitude (Table 2).

The PRPD morphology divides the ageing process into three recognisable stages. In the early stage, the patterns at all frequencies are similar and compact. In the middle stage, discharges above 0.25 V multiply and the patterns begin to stratify, with a high-density discharge cluster emerging near (190°, 0.3 V) at 20 kHz and cluster centroids shifting upward with frequency. In the late stage, the patterns at all frequencies develop a cluster structure in the 320–360° phase window, and quantifying that window on the pattern panels in Figure 4 (occupied-area share and 99th-percentile amplitude reach, Table 2) makes the frequency dependence explicit. At 20 kHz, the finger signature is unambiguous and late-stage specific: the window share jumps from 5.5 to 13.0% and the reach increases from 0.50 to 0.87 V only in the late panel. At 40 kHz an elongated window cluster is already visible in the middle-stage panel (7.6%, 0.43 V) and intensifies in density towards flashover rather than appearing anew. At 30 kHz, the late-stage form stays low in amplitude (0.29 V) on the recorded one-minute panels. The negative half-cycle peak magnitude first grows then shrinks while the positive half-cycle keeps growing. The late-stage finger pattern therefore provides a candidate flashover precursor whose expression is frequency-dependent and sharpest at 20 kHz (Figure 4).

A count-aware companion indicator complements the geometric finger score. The stage statistics in Figure 3 carry both the mean discharge amplitude *A* and the mean repetition number *N* per observation window; trapezoidal integration over the ageing progress axis yields the cumulative discharge energy proxy $C_{\mathrm{eng}}\left(p\right)=\int_{0}^{p}A{\left(u\right)}^{2}\;N\left(u\right)\;du$ (amplitude squared to scale with per-discharge energy, count-weighted). At the end of ageing (p=100%), the terminal value is 9.01, 5.31 and 6.20 at 20, 30 and 40 kHz, respectively; normalised by the observed constant-stress lifetime at that frequency (Table 1), the cumulative activity per unit time $C_{\mathrm{eng}}\left(100\right)/L$ is $1.36\times 10^{-3}$, $1.53\times 10^{-3}$ and $4.03\times 10^{-3}\;V^{2}\cdot \mathrm{count}\cdot s^{-1}$ for 20, 30 and 40 kHz. The terminal integral records the accumulated activity over a complete ageing trajectory, whereas $C_{\mathrm{eng}}\left(100\right)/L$ expresses its mean rate in physical time. Together, they provide a count-aware, non-geometric companion to the finger signature: the accumulated values are 9.01, 5.31 and 6.20, while the corresponding activity rate increases across 20, 30 and 40 kHz. Because the rate uses the observed lifetime as its denominator, it serves as a descriptive time-normalised companion to the accumulated activity.

### 4.3. An Operational Reading of the Three Stages

The preceding statistical and morphological analyses compose into a watch sequence of escalating specificity for an online monitor. Its earliest statistical precursor is the crossover of two summary statistics, a mean magnitude that rises through a falling repetition number, placed by the data at 60–80% of the consumed life across the three complete trajectories and requiring only two numbers per observation window to detect. Its terminal marker is morphological: late-stage occupation or intensification of the finger cluster in the 320–360° window, sharpest at 20 kHz; at 40 kHz, the cluster emerges by the mid-stage and intensifies towards flashover (Table 2). Their frequency-dependent expression, documented in the same table, provides successive warning and terminal-stage observations along the ageing trajectory. At 40 kHz, the interval between the statistical crossover and flashover is measured in minutes rather than hours, motivating shorter PRPD polling intervals as drive frequency rises. In practice, entry into the warning state can trigger denser PRPD sampling, electrical derating or a scheduled inspection before the terminal-stage pattern develops.

### 4.4. Damage Morphology

The optical record shows the damage zone evolving from an arrow-shaped white mark in the early stage to a mallet-shaped erosion zone before flashover, with the white zone extending towards the ground electrode as the discharge channel concentrates; no dendritic tracking of the kind canonical under power-frequency ageing is observed at any test frequency. Surface flashover was accompanied by transient intense burning of the PI surface. The progressive white-mark damage records local surface degradation under the combined electrical and thermal stress.

Table 3 condenses the three stages across the statistical, morphological and optical channels.

## 5. Mechanism and Discussion

Both the frequency dependence and the observed damage morphology can be interpreted consistently through residual surface charge that does not dissipate fully between successive discharges. The residual field left on the surface after a discharge can be represented at first order by

(1) $$E_{\mathrm{res}}=E\left(0^{+}\right)\;\exp\left(-t/\tau \right)$$

where the effective time constant τ summarises a relaxation spectrum set by the bulk and surface conductivities, permittivities and system geometry [15]. Pulsed-electroacoustic observations of frequency-dependent bulk-charge profiles in PI provide independent evidence that the electrical-stress timescale controls charge retention and field redistribution [7]. When the half-cycle interval is long relative to the effective charge-relaxation time, the surface can partially reset between discharges and activity remains spatially dispersed. Raising the frequency into the tens-of-kilohertz range shortens that interval without changing the fixed material and geometry; residual charge accumulates from cycle to cycle and progressively biases subsequent discharges onto an already-formed channel, a mechanism embedded in partial-discharge modelling for decades through the residual charge each event deposits [16]. The consequences line up sequentially. Inception is a local, single-discharge threshold and remained frequency-invariant in the measurements. Ageing, on the other hand, accelerates through two multiplicative channels. The first is a cycle-rate channel: the number of stress cycles per unit time scales with *f* and by itself would yield a strict inverse lifetime law. The second is a per-cycle aggravation channel that switches on once the inter-cycle interval drops below τ: each subsequent discharge then fires into the residual field of its predecessors and lands on a channel that is progressively more conductive and thermally more loaded. The product of these two channels is a lifetime exponent above unity, and the fitted n=2.07 in Section 3 is consistent with the combined action of both channels across 20–40 kHz. The same reading covers both ends of the frequency axis. That the censored 10 kHz lifetime rises above even the power-law extrapolation is consistent with the inter-cycle interval exceeding τ there and the aggravation channel switching off; the right-censored observation supplies a lower bound on the low-frequency lifetime. The discharge channel concentrates rather than branches, hence the linear, mallet-terminated damage track. The positive-half-cycle bias of the late-stage finger clusters is consistent with residual-charge-mediated coupling between charge accumulation and subsequent partial-discharge (PD) activity under converter-type waveforms [17]. Recent external studies provide complementary mechanistic anchors. Whole-life tests on Nomex relate discharge magnitude, pulse count and microscopic erosion to successive ageing stages [18]. At the material interface, recent PI/PEN experiments identify distinct roles for defect-driven inception and surface-charge-controlled extinction [19], while direct surface-charge-decay measurements show that geometry and surface/bulk conductivity jointly set the relaxation rate [15]. Together, these results support resolving the complete trajectory and interpreting the present lifetime, magnitude, repetition and phase-window evolution as complementary rather than interchangeable observables.

For application, the results translate into two rules. For insulation design of HFPT/SST, surface-insulation life budgets shrink roughly with the square of the operating frequency over the 20–40 kHz band, and creepage distances that are conservative at power frequency are not automatically conservative at 40 kHz. For condition monitoring, the finger-like PRPD signature is a candidate flashover precursor on PI surfaces, sharpest at 20 kHz and frequency-dependent in expression within this experiment (Table 2). A two-stage monitoring rule follows from the recorded trajectory: the magnitude–repetition crossover at 60–80% of normalised life provides the warning state, and subsequent late-stage occupation or intensification of the 320–360° finger window provides the candidate device-level morphological criterion. Both quantities are available from phase-resolved magnitude statistics already produced by standard PD monitors. The fitted relation also gives the designer a first-order translation along the frequency axis: migrating a surface-insulation system from 20 to 40 kHz divides its expected life by $2^{2.07}\approx 4.2$ under the conditions tested here, so an endurance qualification performed at the lower frequency would, on this point estimate, need to run about 4.2 times longer or demonstrate a corresponding stress margin to support the same service life at the higher one. For design margin, the observed pairwise-exponent range of 1.6–2.8 provides an envelope around the point estimate. The five-run inception and flashover measurements show a run-to-run spread of ±0.2–0.9 kV (Table 1), supporting the frequency trend independently of the lifetime fit.

### Relation to Prior High-Frequency Surface-Discharge Work

Four lines of prior work frame this study. The converter-endurance line [17] stresses machine and film insulation with repetitive impulsive or pulse-width-modulated (PWM) waveforms, where frequency enters through the pulse repetition rate and endurance is reported against stress amplitude. Closest on the material axis, polyimide lifetime under partial-discharge ageing has been resolved against temperature, pressure and humidity [20]; a controlled sinusoidal drive-frequency axis with all other conditions fixed is part of neither design. The mechanism line [9,21,22,23] resolves charge transport, streamer propagation and flashover physics on solid surfaces, but does not quantify insulation life at solid-state transformer frequencies. High-frequency PI surface-discharge studies map influencing factors and stage transformation, providing a complementary morphology baseline [10,11]. Against this broader background, the present study fits the measured frequency-dependent lifetime results with an inverse power law (exponent n=2.07 with $R^{2}=0.97$, Section 3); the 320 to 360 degree window is scored on a nine-cell PRPD table (share and reach, Table 2); and the crossover of mean amplitude and mean repetition number is located at the 60 to 80 % ageing-progress point across the three frequencies that reached flashover (Section 4). Recent lifetime studies treat voltage rise rate and high-frequency steep-pulse waveform as distinct acceleration axes [4,24]; the grid here instead isolates sinusoidal drive frequency. Table 4 summarises the comparison.

## 6. Conclusions

Across 20 to 40 kHz, the electrode system, the specimen batch and the protocol were held fixed while only the driving frequency moved, so the frequency axis of PI surface discharge could be read directly. The contributions are threefold. (a) Firstly, frequency resolved lifetime quantification: surface discharge life falls as an inverse power law with exponent near two, supported by a censored low frequency bound. (b) Stage resolved PRPD signatures, including a finger cluster in the 320 to 360 degree window preceding flashover, most articulated at 20 kHz and quantified on the recorded patterns. (c) Finally, a space charge interpretation was proposed linking the per-cycle charge injection budget growth at high frequency to the suppressed inter-cycle dissipation, consistent with both the lifetime relation and the non-dendritic damage morphology.

The quantitative lifetime relation informs the insulation design of high-frequency transformers, and the finger signature provides online monitors with a candidate alarm criterion, sharpest at 20 kHz, for polymer surface insulation.

## Figures and Tables

**Table 1:** Inception voltage and flashover voltage (five-run mean ± run-to-run spread across n=5 replicates per frequency) and constant-stress (12 kV) surface lifetime of PI versus frequency. Inception voltage is frequency-invariant within the observed run-to-run spread of ±0.2–0.4 kV. ^†^ Right-censored: no flashover was observed within the 10 h test window.

Frequency (kHz)	10	20	30	40
Inception voltage (kV)	≈3.8	≈3.8	≈3.8	≈3.8
Flashover voltage (kV)	18.4±0.9	18.1±0.5	16.9±0.6	16.1±0.3
Lifetime at 12 kV (s)	>$3.6\times 10^{4}$†	6608	3480	1536

**Table 2:** Quantification of the 320–360° window on the nine PRPD panels of Figure 4: share of the pattern’s occupied area falling in the window and 99th-percentile amplitude of that area (share %/reach V).

Frequency	Early	Middle	Late
20 kHz	5.5/0.33	5.5/0.50	13.0/0.87
30 kHz	2.0/0.33	1.4/0.32	3.1/0.29
40 kHz	1.1/0.27	7.6/0.43	8.4/0.44

**Table 3:** Three-stage synthesis of the discharge record: summary statistics, phase-resolved morphology, optical damage development and the monitoring role of each stage. Entries condense the observations of this section.

Quantity	Early Stage	Middle Stage	Late Stage
Mean discharge magnitude	near 0.04 V, rising	rising; discharges above 0.25 V multiply	positive half-cycle magnitude keeps growing while the negative peak first grows then shrinks
Repetition number per window	rising	peaks at 60–80% of consumed life (6.9–9.8 across frequencies)	falling: fewer but stronger discharges
PRPD morphology	compact, similar across frequencies	stratifying; high-density cluster near (190°, 0.3 V) at 20 kHz, centroids shifting upward with frequency	finger-like clusters in the 320–360° window, most articulated at 20 kHz (Table 2)
Damage zone (optical record)	arrow-shaped white mark	erosion zone concentrating and extending towards the ground electrode	mallet-shaped erosion zone ending in flashover; no dendritic tracking at any frequency
Monitoring role	baseline	statistical precursor: magnitude–repetition crossover	terminal marker: late-stage occupation or intensification of the finger pattern (expression frequency-dependent, Table 2)

**Table 4:** This study against neighbouring lines of work. Line sources, top to bottom: converter endurance [17]; PI ageing under PD [20]; surface-discharge physics [9,21,23]; HF transformer insulation [10,11].

Line	Frequency Treatment	Life Quantification
Converter endurance	pulse repetition rate of PWM stress	endurance vs. stress amplitude
PI ageing under PD	environment (T, p, RH), fixed drive	life vs. environment
Surface-discharge physics	single condition or DC/AC contrast	not addressed
High-frequency PI surface discharge	factor analysis and fixed-frequency stage transformation	lifetime scaling not the primary outcome
This work	four sinusoidal frequencies, fixed geometry and stress	power law n=2.07, $R^{2}=0.97$, censored low-frequency bound

## Data Availability

The data presented in this study are available from the corresponding author on reasonable request.

## Abbreviations

The following abbreviations are used in this manuscript:

HFCT	High-frequency current transformer
HFPT	High-frequency power transformer
PD	Partial discharge
PI	Polyimide
PRPD	Phase-resolved partial discharge
PWM	Pulse-width modulation
SST	Solid-state transformer
